# Orthogonal Experimental Optimization of Preparation and Microstructural Properties of a Diffusion Barrier for Tantalum-Based Silicide Coatings

**DOI:** 10.3390/ma16114097

**Published:** 2023-05-31

**Authors:** Lairong Xiao, Jiawei Xu, Xiaojun Zhou, Yafang Zhang, Guanzhi Deng, Hongtai Shen, Wei Li, Xiaojun Zhao, Zhenyang Cai

**Affiliations:** 1 School of Materials Science and Engineering, Central South University, Changsha 410083, China; xiaolr@csu.edu.cn (L.X.); csuxujiawei@csu.edu.cn (J.X.); csuczy@csu.edu.cn (Z.C.); 2 Key Laboratory of Non-Ferrous Metal Materials Science and Engineering, Ministry of Education, Central South University, Changsha 410083, China; 3 State Key Laboratory of Powder Metallurgy, Central South University, Changsha 410083, China

**Keywords:** tantalum alloy, silicide coating, diffusion barrier, TaB_2_, TaC, orthogonal experiment

## Abstract

To solve the problem of silicide coatings on tantalum substrates failing due to elemental diffusion under high-temperature oxidation environments and to find diffusion barrier materials with excellent effects of impeding Si elemental spreading, TaB_2_ and TaC coatings were prepared on tantalum substrates by the encapsulation and infiltration methods, respectively. Through orthogonal experimental analysis of the raw material powder ratio and pack cementation temperature, the best experimental parameters for the preparation of TaB_2_ coatings were selected: powder ratio (NaF:B:Al_2_O_3_ = 2.5:1:96.5 (wt.%)) and pack cementation temperature (1050 °C). After diffusion treatment at 1200 °C for 2 h, the thickness change rate of the Si diffusion layer prepared using this process was 30.48%, which is lower than that of non-diffusion coating (36.39%). In addition, the physical and tissue morphological changes of TaC and TaB_2_ coatings after siliconizing treatment and thermal diffusion treatment were compared. The results prove that TaB_2_ is a more suitable candidate material for the diffusion barrier layer of silicide coatings on tantalum substrates.

## 1. Introduction

As a widely used high-temperature structural material in the aerospace industry in recent years, tantalum metal and its alloys have broad development potential and research value [1,2,3,4,5]. However, although tantalum possesses good physical and chemical properties [6,7], it is highly susceptible to oxidation failure at high temperatures [8,9,10]. Its high-temperature oxidation resistance needs to be enhanced to meet high-temperature conditions. To solve the problem of poor oxidation resistance of tantalum and its alloys in high-temperature environments, high-temperature oxidation-resistant coatings are often applied to extend the service life of the material [11,12,13]. Current antioxidant coatings for tantalum and its alloys include oxide coatings, noble metal coatings, aluminide coatings, and silicide coatings [14,15,16,17,18]. Among them, researchers at home and abroad have extensively studied silicide coatings due to their broad scope of use and easy modification and optimization properties [11,19,20,21,22]. A series of significant research results has been achieved, forming a series of more mature silicide coating systems. The service life of antioxidant coatings is not only related to their antioxidant capacity but also needs to take into account possible abnormal failures during service, such as “pesting” pulverization [23,24], volatilization of low-melting-point components [25], loss of antioxidant elements due to interdiffusion of high-temperature elements [26,27,28], and Kirkendall holes [29]. However, most research on the modification of silicide coatings is focused on enhancing the antioxidant properties of the coating itself through a series of doping methods [30,31]. In contrast, research on extending the coating life by changing the coating structure and suppressing the interdiffusion of high-temperature elements is relatively rare [32].

Among the compounds of refractory metals, borides and carbides have high melting points (≥2500 °C) and strong covalent bonding, with good structural stability in high-temperature environments; furthermore, their coefficient of thermal expansion is close to that of refractory metals, with a high lattice density [33,34,35], which helps to inhibit the diffusion of Si atoms in ultra-high-temperature environments. The diffusion energy barrier of Si atoms in the TaB_2_ lattice (0.43 eV) is much greater than that in the Ta lattice (0.0359 eV), as evidenced by first-principles calculations, and the effectiveness of TaC as a barrier to Si diffusion has been confirmed in several studies [36,37,38]. Therefore, borides and carbides are justified as potential diffusion barrier materials. Since few studies have reported on the preparation of boron infiltration layers on tantalum substrates via the over-dipping process, the experimental design is insufficient for reference targets. To ensure the stability and controllability of the boriding process, optimization of the process parameters of the pack cementation by orthogonal experimental design can be used to identify more suitable process parameters quickly and effectively.

In this work, the microstructure of boride and carbide diffusion barriers was studied, and its practical application value was evaluated. Orthogonal experiments were designed for the process of boronizing on tantalum substrates to obtain stable process parameters; the relevant diffusion barrier layers were prepared on tantalum substrates via the process of pack cementation, and the two diffusion barriers were subjected to siliconizing treatment under the same process conditions, followed by heating to 1200 °C and holding for 2 h. The evolution of the structure during siliconizing and thermal diffusion was investigated.

## 2. Materials and Methods

### 2.1. Sample Preparation

Pure tantalum (99.8% purity, Baoji Junuo Metal Material Co., Ltd., Baoji, China) was used as the base material in the experiment. Strip samples (with dimensions of 70 mm × 7 mm × 2 mm) were cut from a pure tantalum block. The samples were ground with SiC grit papers, followed by pickling and alkali washing; finally, all samples were cleaned in an ultrasonic ethanol bath. 

The carbide and boride diffusion barrier embryos on tantalum substrates were then prepared by the following steps:

1. Carbide diffusion barrier:

The carbide barrier layer on a tantalum substrate was obtained through carburization treatment. Carbon black powders were dried at 70 °C for 2 h and placed in an alumina corundum crucible, into which the tantalum substrate samples were dispersed and buried. The corundum crucible was placed in a high-temperature tube furnace and heated to 1000 °C in an argon atmosphere (0.8 atm) for 10 min, then furnace-cooled to room temperature. The surface of the specimens were polished and cleaned with alcohol for 5 min to obtain a clean surface.

2. Boride diffusion barrier:

B powders (99.9% purity, 1μm, Sinopharm Chemical Reagent Co., Ltd., Shanghai, China), filler (Al_2_O_3_) (99% purity, 5 μm, Tianjin kemiou chemical reagent Co., Ltd., Tianjin, China), and activator (NaF) (98% purity, 2 μm, Tianjin kemiou chemical reagent Co., Ltd., Tianjin, China) were mixed in a particular ratio to form boronizing powders. The boronizing powders were mixed at 150 r/min for 12 h, then dried at 70 °C for 2 h. The powders were poured into an alumina corundum crucible, into which the tantalum substrate samples were dispersed and buried. The corundum crucible was placed in a high-temperature tube furnace and heated to 1050 °C for 1 h, then furnace-cooled to room temperature.

**Figure 1:**
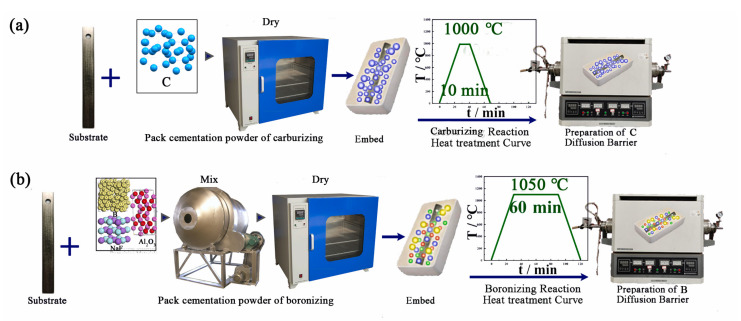
Schematic diagram of the pack cementation process: (**a**) carburizing process; (**b**) boronizing process.

A flow chart of the process is shown in Figure 1.

### 2.2. Orthogonal Experiment of the Boronizing Process

A three-variable, three-level orthogonal experimental design was used. The three variables were NaF content, B powder content, and pack cementation temperature. The three levels are NaF content were 2.5 wt.%, 5 wt.%, and 7.5 wt.%. The three levels of B powder content were 1 wt.%, 2 wt.%, and 3 wt.%. The three tested pack cementation temperatures were 1050 °C, 1100 °C, and 1150 °C. The above parameters were formulated with reference to the process parameters of a B diffusion barrier layer prepared on Nb and Mo by our research group in the earlier stage and relevant literature [39,40]. According to the parameters, the L9 orthogonal experimental table was selected, as shown in Table 1.

### 2.3. Microstructural Characterization

X-ray diffraction (XRD, Rigaku D/Max 2500, Rigaku, Tokyo, Japan) with Cu Kα radiation at 40 kV and 250 mA was used to analyze the phase and crystallinity of the coating surface. A scanning electron microscope (SEM, FEI Sirion 200, FEI, Hillsboro, OR, USA) equipped with an energy-dispersive spectrometer (EDS) was used to analyze the microstructure and morphology of the surface and interface of the coating material and to analyze the composition of the local phase. An electron probe microanalyzer (EPMA, JEOL JXA-8230, JEOL, Tokyo, Japan) equipped with a wavelength-dispersive spectrometer (WDS) was used to analyze the micro-area composition of the coating surface and cross section.

### 2.4. Blocking Diffusion Performance Test

The effects of carbide and boride diffusion barriers on the diffusion-resistant performance of the Si element was investigated. Both diffusion barriers were subjected to siliconizing and thermal diffusion treatment. First, all samples were embedded in a homogeneous mixture of Si powders, Al_2_O_3_ powders, and NaF powders, then held at 1250 °C in an argon atmosphere (0.8 atm) for 2 h. Secondly, the thermal diffusion treatment was carried out on the samples after direct siliconizing in the crucible and holding them in a tube furnace at 1200 °C in an argon atmosphere (0.8 atm) for 2 h.

## 3. Results and Discussion

### 3.1. Results and Analysis of Orthogonal Experiment of Boronizing Process

Table 2 shows the range analysis of the orthogonal test results of the embedding boronizing process. In the table, K1, K2, and K3 are the sums of the test results of the three levels of the factors in the column, while k1, k2, and k3 are the average values of the thickness of the boronizing layer at three levels of each influencing factor, which reflect the influence of different levels of a given factor on the thickness of the boronizing layer. Range (R) is the difference between the maximum value and the minimum value in k1, k2, and k3, which roughly reflects the significance of the influence of the level change of a given factor on the thickness of the boronizing layer; the importance of the effect is proportional to the size of the range. The ranges of NaF content, B content, and pack cementation temperature were 18.03, 48.8, and 5.93, respectively. Accordingly, we can rank the significant degree of influence of three factors on the boronizing layer thickness from large to small as follows: B content, NaF content, and pack cementation temperature. As a standard, k1, k2, and k3 of the three factors were compared. For example, the average values of the thickness of the boronizing layer at three levels of B content were 25.26, 51.93, and 74.07, respectively. In other words, when the B content was 3%, the boronizing layer was the thickest, and when the B content was 1%, the boronizing layer was thinner. Similarly, when the content of NaF was 7.5%, the boronizing layer was the thinnest, and when it was 5%, the boronizing layer was the thickest. The boronizing layer was the thinnest when the encapsulation temperature was 1100 °C and the thickest when the temperature was 1150 °C, but there was little difference between the three temperatures.

The theoretical basis of using NaF as an activator for boronizing is that NaF reacts with B as follows at high temperatures (727 °C–1227 °C) [41]:

B + xNaF → BF_x_(g) + Na(g) (x = 1, 2) (1)

Driven by the potential chemical gradient, the BFx gas generated by the reaction adsorbs on the surface of the substrate, and then the active atoms caused by the subsequent reaction [B]:

(x + 1)BF_x_(g) → [B] + xBF_x+1_(g) (x = 1, 2) (2)

The active boron atom reacts with the substrate and diffuses gradually:

B + Ta → TaB_2_ (3)

**Figure 2:**
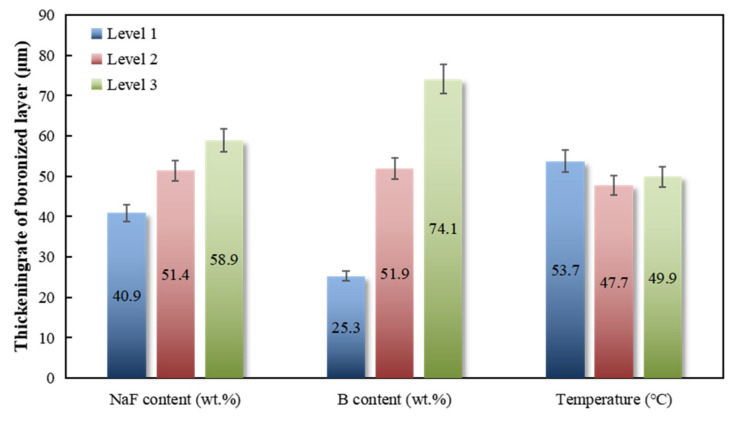
Influencing factors relative to changes in the average thickness of the boriding layer.

The reaction of boronization can be divided into three steps: gas generation, transport and adsorption, and reaction diffusion. The above reaction was thermodynamically simulated, and we found that the generation of BF_x+1_(g) did not vary significantly with temperature in the range of 727 °C–1227 °C, and the change in free energy in reaction (3) in the range of 0–1250 °C was only 2.82 kJ/mol after thermodynamic calculation. This shows that although the temperature change affects the embedding boronizing process, it is not sensitive. As shown in Figure 2, the temperature change causes the thickness of the boronizing layer fluctuate in a small range. NaF participates in the reaction as an activator in the process of encapsulation, and its main role is to generate BFx(g) and transport it to the surface of the substrate to create active boron atoms. The consumption rate of active boron atoms depends on the gas adsorption on the substrate surface and the subsequent reaction and diffusion. Since the partial pressure of the gas phase generated by reaction (1) is constant at a specific temperature, it has a strong diffusion and transport capacity at high temperatures, and with a particular substrate surface area, the marginal benefit of increasing NaF concentration after the amount of BFx is sufficient to fully cover it gradually decreases, as shown in Figure 2. With the increase in NaF content, the thickening rate of the boronized layer decreases. The overall rate of this process not only depends on the actual reaction rate of a certain reaction but also on the diffusion process of each substance when transported from its original position to the reaction position. Although the gas phase generated by reaction (1) dramatically improves the deposition efficiency and transport efficiency of B on the substrate surface, B atoms closer to the surface are still preferentially consumed. Increasing the concentration of boron atoms can increase the number of boron atoms in the overall region, resulting in more boron atoms in close range. While the transport rate remains unchanged, it can effectively reduce the transport distance and thereby improve the overall reaction speed. This effect can significantly increase the thickness of the diffusion layer when the concentration of boron atoms is not high. However, as the concentration of boron atoms increases, the reaction rate of reaction (1) or the transport efficiency of the gas-phase substances it produces can act as a constraint on its rate, resulting in a gradual decrease in the effect of increasing the concentration of B atoms on the thickness improvement, as shown in Figure 2.

Table 3 shows the variance analysis of the orthogonal test results of embedding boronizing. Among the three influencing factors, only the content of B shows a significant change, followed by the content of NaF. However, within the range of 1050 °C–1150 °C, the temperature change has almost no effect on the thickness of the boronizing layer. According to relevant studies, excessive diffusion speed leads to interface unevenness and affects the binding of the coating to a certain extent. Ultimately, a powder ratio of NaF:B:Al_2_O_3_ = 2.5:1:96.5 was adopted, the pack cementation temperature was selected as 1050 °C, and the diffusion layer thickness was selected by extending the holding time.

### 3.2. Analysis of the Microstructure of the Diffusion Barrier Layer

**Figure 3:**
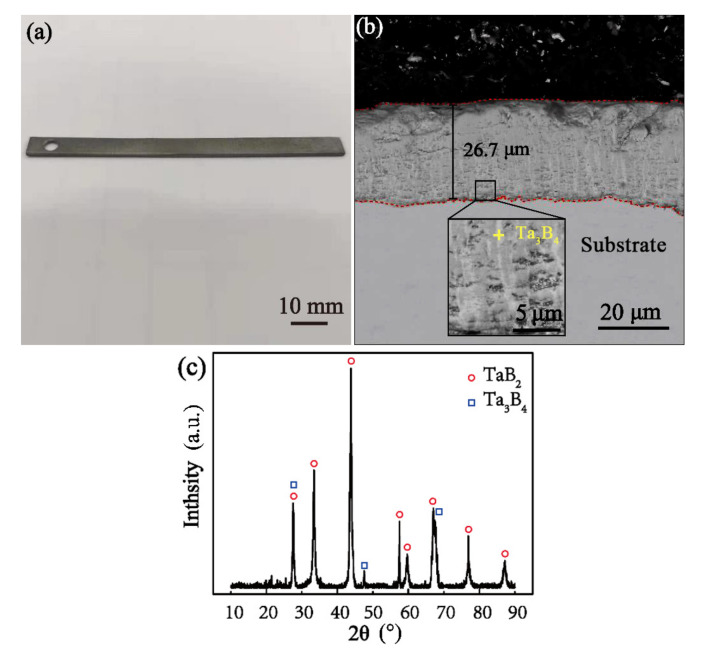
Boride diffusion layer: (**a**) sample macro diagram; (**b**) boronized section topography; (**c**) surface XRD detection after boronizing.

Figure 3 shows the macroscopic surface and cross-sectional morphology of the boride layer. Figure 3a shows a macroscopic diagram of the boronized sample. There is white powder attached to the surface of the boronized layer, which is dark gray after cleaning treatment, with no apparent metallic luster and no obvious macroscopic defects.

As observed by the back-scattered mode of scanning electron microscopy (Figure 3b), the boride layer prepared by the encapsulation process is dense up to 26.7 μm in thickness, but there are light-colored strips at the bottom of the layer. In combination with the characteristics of the pack cementation process and XRD analysis results, as shown in Figure 3c, the low-boride Ta_3_B_4_ of Ta appears at the bottom. It is worth noting that Ta_3_B_4_ is an unstable phase in Ta-B compounds [35], so this phase was not found again in the subsequent detection.

**Figure 4:**
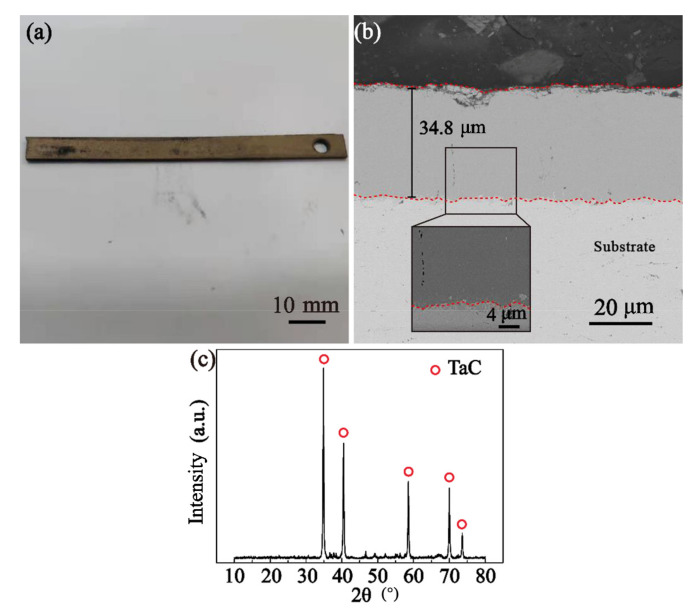
Carbide diffusion layer: (**a**) sample macro diagram; (**b**) carburized section topography; (**c**) surface XRD detection after carburizing.

Figure 4 shows the macroscopic surface and cross-sectional morphology of the carbide layer. In Figure 4a, the surface of the carbide layer is pale gold, with obvious metallic luster after treatment and an overall level and smooth surface. According to the SEM image in Figure 4b, the thickness of the coating can reach 34.8 μm, and the overall coating is more uniform and compact. However, point defects perpendicular to the carbonized layer can be observed in local areas. XRD analysis shows that the main phase of the carbide layer in Figure 4c is TaC. Combined with the phase diagram [34], it can be seen that there is a large solid solubility between TaC and C, and these continuously distributed point defects may be traces left by the rapid diffusion of carbon atoms in TaC.

### 3.3. Structural Evolution of Diffusion Layer Siliconizing Treatment and Thermal Diffusion

To further optimize the structure of the diffusion barrier, three different thicknesses of the boride diffusion barrier and carbide diffusion barrier were designed, and siliconizing treatment was carried out under the same process conditions.

**Figure 5:**
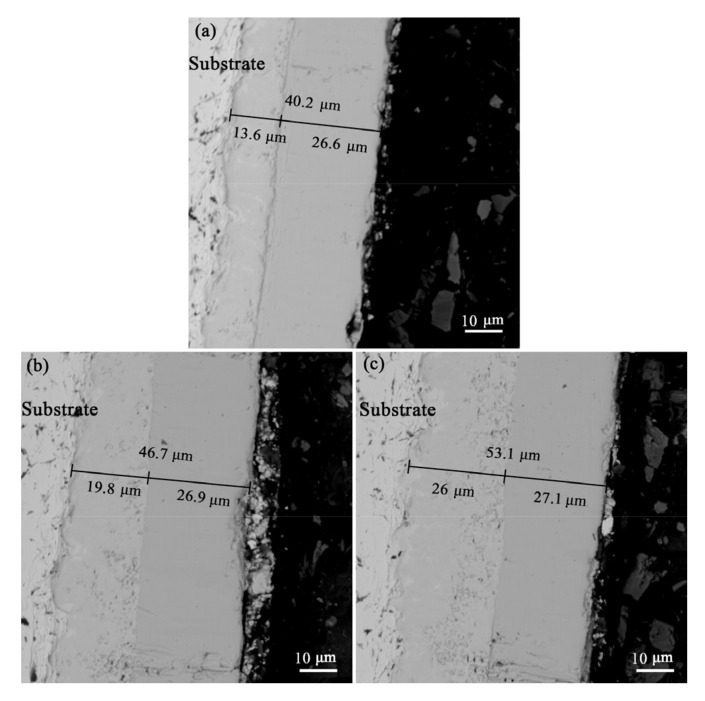
Siliconized cross section of the boronized layer with three thicknesses: (**a**) boronized for 0.5 h; (**b**) boronized for 1 h; (**c**) boronized for 1.5 h.

As shown in Figure 5, the boride layer thickness of the three coatings was regulated by different pack cementation times (0.5 h, 1 h, and 1.5 h), with thicknesses of 10.2 μm, 19.7 μm, and 26 μm, respectively, and the siliconizing treatment was uniformly carried out at 1250 °C for 2 h. The overall structure of the diffusion layer can be divided into two layers: the boron diffusion layer near the substrate and the silicon diffusion layer outside. The total thickness of the diffusion layer increased successively, and the thickness of the silicon diffusion layer was 26.6 μm, 26.9 μm, and 24.4 μm, respectively. The difference was slight, mainly resulting from the boron diffusion layer. In addition, the thickness of the diffusion barrier layer of the three coatings changed little before and after siliconizing, and the overall thickening was mainly due to the appearance and thickening of the silicon diffusion layer. The reason for this is that in the early stage of siliconizing, these three types of boron diffusion barrier layers had sufficient ability to hinder diffusion, so Si atoms could not penetrate the boron diffusion layer. As Si diffused inward, B element also continued to diffuse into the substrate, resulting in advancement of the boron diffusion layer inward, with little change in thickness. The overall coating was compact and well-bonded, with some cavity defects at the interface of the silicon diffusion layer and the boron diffusion layer, leaving traces of rapid diffusion.

**Figure 6:**
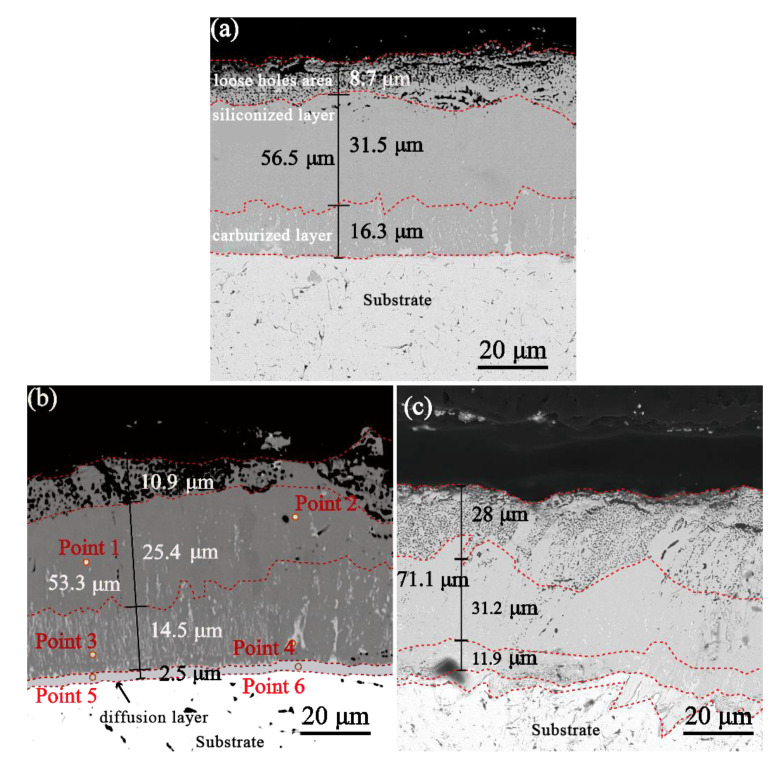
Siliconized cross section of the carburized layer with three thicknesses: (**a**) carburized for 10 min; (**b**) carburized for 30 min; (**c**) carburized for 1 h.

Figure 6 shows the sample cross sections after carburizing treatment for 10 min, 30 min, and 1 h and siliconizing at 1250 °C for 2 h. The three groups of coatings all exhibit multilayer structures. The samples carburized for 10 min are divided into a silicon layer and a carburized layer from the surface to the inside, with a ′loose holes area′ on the surface of the siliconized layer (Figure 6a), while the samples carburized for 30 min and 1 h have a secondary diffusion layer in the innermost part on this basis (Figure 6b,c). Among them, the secondary diffusion layer of the sample carburized for 30 min is more uniform (Figure 6b), while the sample carburized for 1 h shows a partially raised interface morphology (Figure 6c). The reason for the appearance of the “loose holes area” is speculated to be that during the siliconizing process, Si element in the early stage has a fast inward diffusion rate, and the diffusion coefficients of the inner and outer layers are quite different from each other, leading to the appearance of holes on the surface of the coating. With the progress of diffusion, the carbon diffusion layer loses the solid solution C atom, and the Si atom increases. This reduces the difference between the internal and external diffusion coefficients, resulting in a slower diffusion rate, so there are no more holes in the interior. The total coating thicknesses of the three groups were 56.5 μm, 53.3 μm, and 71.1 μm, respectively. The thicknesses of the siliconized layers were 40.2 μm, 36.3μm, and 59.2 μm, respectively. The thicknesses of the carburized layers were 16.3 μm, 14.5 μm, and 11.9 μm, respectively. The diffusion layer of the sample carburized for 10 min was extremely thin and almost invisible (Figure 6a). The diffusion layer thickness of the sample carburized for 30 min was about 2.5 μm (Figure 6b). However, the diffusion layer interface of the sample carburized for 1 h was extremely uneven, and the layered structure was lost to a certain extent. This phenomenon may have been caused by the uneven internal diffusion of surplus C atoms in the solid solution in the TaC phase during the subsequent siliconizing process with the extension of the encapsulation time. Combined with EPMA point composition analysis (Table 4), the main components of the siliconized layer can be inferred from atomic proportions to be TaSi_2_ and TaC. The proportion of silicon in the carburized layer decreased significantly (<10%). The proportion of silicon in the transition layer at the bottom was further reduced. According to the Ta-C phase diagram, the main composition is a mixed phase of TaC and TaC_1−x_.

**Figure 7:**
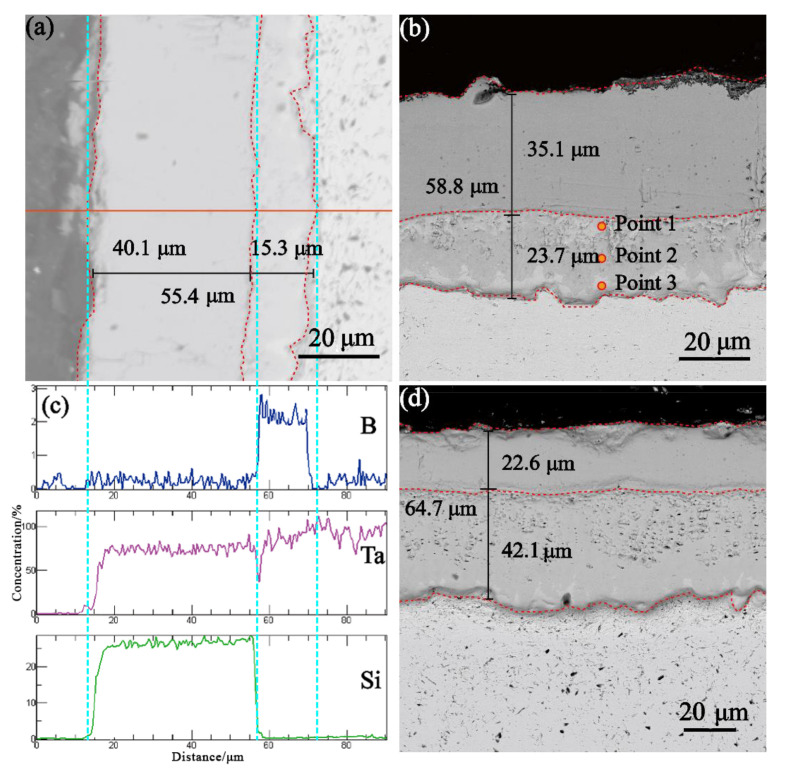
Evolution of the cross-sectional morphology of the B-Si diffusion layer after thermal diffusion treatment: (**a**) cross section of sample boronized for 0.5 h; (**b**) cross section of sample boronized for 1 h; (**c**) line scan analysis of a sample section after boronization for 0.5 h; (**d**) cross section of sample boronized for 1.5 h.

To investigate the blocking effect of the boride diffusion layer and carbide diffusion layer with different thicknesses on silicon diffusion, six groups of original samples were placed a tube furnace for thermal diffusion treatment at 1200 °C for 2 h. Since surface oxidation changes the thickness of the silicon diffusion layer to a certain extent, affecting subsequent analysis, Ar gas flow was used to protect the diffusion process. The evolution of the cross-sectional morphology of the B-Si diffusion layer after thermal diffusion at 1200 °C for 2 h is shown in Figure 7, and the thickness change data of each layer are shown in Table 5, in which “Thickening rate” indicates the proportion of thickness variation in the original thickness and “Thickening proportion” indicates the proportion of thickness variation of the B/Si diffusion layer in the aggregate thickness variation. The overall structure of the samples boronized for 0.5 h and 1 h was still intact and compact (Figure 7a,b), but the samples boronized for 1 h and 1.5 h had hole defects at the top of the boron diffusion layer (Figure 7b,d), and the defect density of the samples boronized for 1.5 h was relatively high (Figure 7d). In addition, the total thickness of the diffusion layer increased to different degrees (Table 5), with a minimum increase of 11.6 μm for the sample boronized for 1.5 h and a maximum increase of 15.2 μm for the sample boronized for 0.5 h, which is in the same order as the thickening rate. The increase in sample thickness after boronizing for 0.5 h and 1 h was mainly due to the growth of the silicon diffusion layer, accounting for 88.2% and 67.77% of the total thickness of the diffusion layer, with increased thickness of 13.5 μm and 8.2 μm, respectively. It can be concluded that the B diffusion layer migrated inward as a whole. However, in the samples boronized for 1.5 h, the increase in diffusion layer thickness was entirely the result of the B diffusion layer, and the silicon diffusion layer became thinner, which indicates that B atoms diffuse in both directions at the same time, which leads to the bidirectional growth of the B diffusion layer. In addition, it can be observed that the thickening rate of the B diffusion layer is directly proportional to its thickness.

From the perspective of composition distribution, the EDS line scan (Figure 7c) show that the silicon element is effectively blocked outside the boron diffusion layer, while the boron element is mainly distributed in the boron diffusion layer, with no apparent signs of diffusion. Furthermore, compared with the original sample, the contrast difference in the boundary area of the boron diffusion layer is obvious (it is more evident in the sample boronized for 1 h). According to the point composition analysis of samples boronized for 1 h (Table 6), it can be inferred from the atomic proportion that the main composition in the central area of the boron diffusion layer is still TaB_2_, while the light-colored phase in the boundary area is low-boride, and the whole boron diffusion layer is infiltrated with trace Si atoms. This shows that B atoms in the B diffusion layer diffuse bidirectionally, and the bonding defects are mainly distributed in the upper half of the B diffusion layer. It can be inferred that a large number of holes in the samples boronized for 1.5 h are Kirkendall defects caused by B-Si interdiffusion. In the phase diagram, TaB_2_ and B have high solid solubility due to the bidirectional diffusion of a large number of B atoms dissolved in TaB_2_ along the composition gradient.

In comparison, the sample boronized for 1.5 h had the most substantial inhibition effect on the thickness increase in the Si diffusion layer, but there was a rapid growth of the B diffusion layer accompanied by a large number of Kirkendall defects (Figure 7d). The thermal stress caused by temperature field fluctuation in the service environment may be concentrated here, which is not conducive to the structural stability of the coating. However, the thickening rate of the Si diffusion layer of the sample boronized for 1 h was only 30.48%, which is much lower than that of the sample boronized for 0.5 h (50.75%). In addition, the thickness growth rate of the B diffusion layer was moderate, with only a few Kirkendall defects (Figure 7b), making it an ideal choice among the three groups of samples.

**Figure 8:**
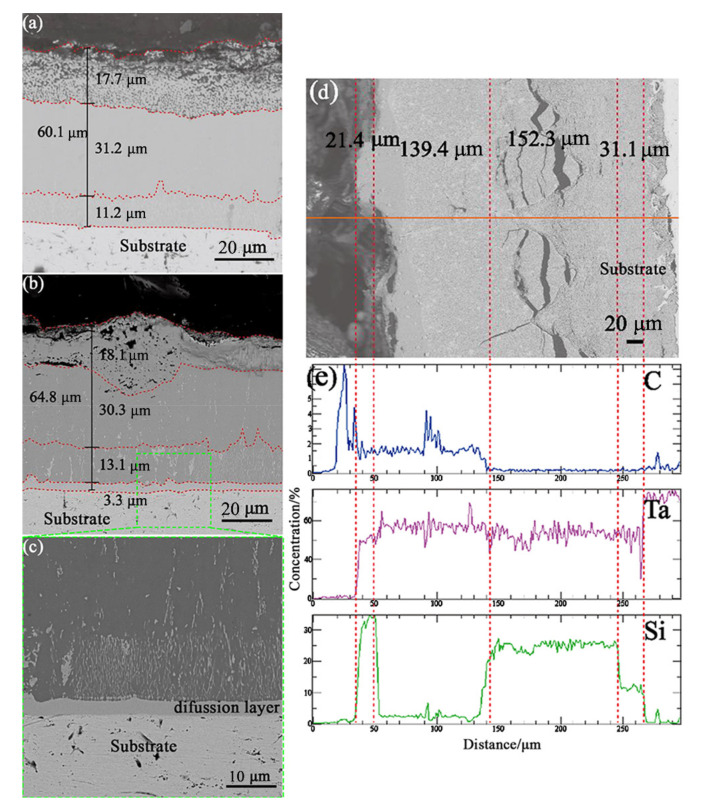
Evolution of the cross-section morphology of the C-Si diffusion layer after thermal diffusion treatment: (**a**) cross-section of sample carburized for 10 min; (**b**) cross-section of sample carburized for 30 min; (**c**) partial enlarged image of (**b**); (**d**) cross-section of sample carburized for 1 h; (**e**) line scan analysis of sample section after carburization for 1 h.

The three groups of carburized samples were subjected to thermal diffusion experiments, and the experimental conditions were the same as those of the three groups of boronized samples. The cross-sectional morphology of the diffusion layer of samples carburized for 10 min and 30 min is shown in Figure 8a–c, and the thickness change is shown in Table 7. The C diffusion layer of two groups of samples was thinned to some extent, with thickening mainly resulting from the silicon diffusion layer. The diffusion layer C was thinned, and the total thickness was not changed obviously, which indicates that C atoms do not have a high internal diffusion, while Si atoms migrate into the diffusion layer C. In addition, the contrast of each layer did not change obviously in the organizational structure. Judging from the thickness change of the siliconized layer, the sample carburized for 10 min was better than the sample carburized for 30 min.

It is worth noting that the porous layer on the surface of the sample carburized for 1 h disappeared, and cracks parallel to the coating interface were produced along the interface (Figure 8d). According to EPMA line analysis, carbon atoms were enriched on the surface to form a carbon diffusion layer with a thickness of about 100 μm, while silicon atoms only formed a carbon–silicon coexistence zone with a thickness of about 10 μm on the surface, with almost no silicon atoms in the middle part of the carburized layer. However, taking the crack at the bottom of the carburized layer as the boundary, the enrichment of silicon atoms began to appear above the crack. Further into the substrate, the proportion of silicon atoms increased rapidly, while carbon atoms almost disappeared. Due to the long diffusion path near the substrate, the supply of silicon atoms was insufficient, and low silicide began to form, which showed a stepped decline in the curve. It can be inferred that the carburized layer, as a diffusion barrier, almost lost its role in blocking the diffusion of silicon atoms.

### 3.4. Comparison and Selection of Diffusion Barrier Layer

**Figure 9:**
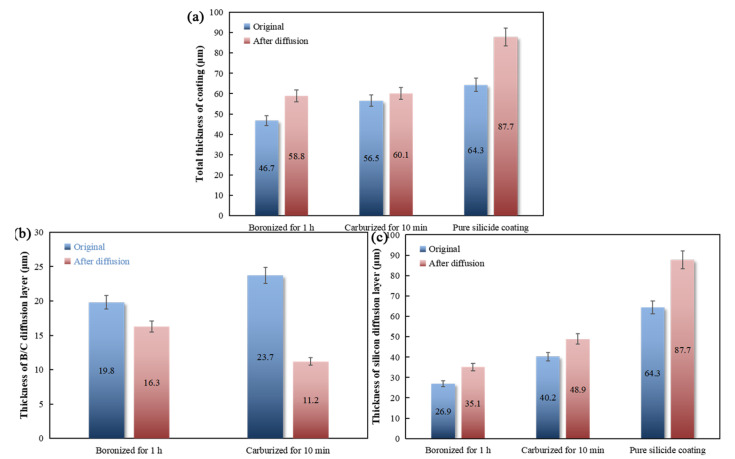
Thickness change of each layer of sample boronized for 1 h and carburized for 10 min and the pure siliconized layer before and after diffusion: (**a**) total thickness change of the coating; (**b**) thickness change of the B/C diffusion layer; (**c**) thickness change of the silicon diffusion layer.

To further compare the two kinds of diffusion barriers, two types of coatings containing diffusion layers were compared with a single Si-Ta coating. The thickness change data of the silicon diffusion layer of samples boronized for 1 h and carburized for 10 min with pure silicide coating were compared, as shown in Figure 9.

**Figure 10:**
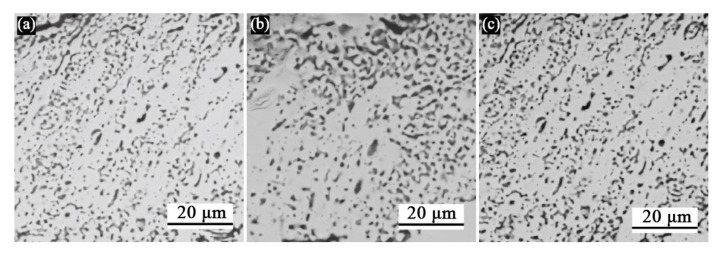
Schematic diagram of the statistical selection area of the porosity of the carburized porous layer: (**a**) sample selection after carburizing for 10 min; (**b**) sample selection after carburizing for 30 min; (**c**) sample selection after carburizing for 1 h.

**Figure 11:**
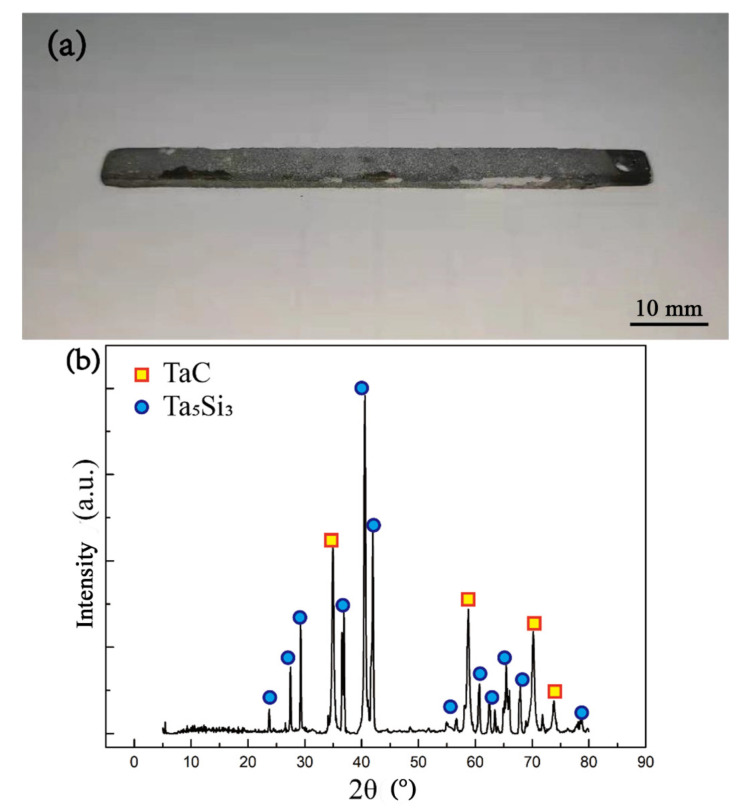
Exfoliated samples and XRD patterns of sample carburized for 10 min after siliconizing treatment: (**a**) original sample; (**b**) XRD pattern of the exfoliated region.

In addition, as mentioned above, before and after diffusion treatment, the outermost layers of the three carburized groups of samples had different degrees of porous structures. According to the statistical analysis, the porosity of the porous areas (Figure 10a, Figure 10b,c) of the three groups of samples before thermal diffusion was 26.2%, 31.3%, and 32.5%, respectively, which is enough to affect the mechanical properties of the materials. After the samples carburized for 10 min were sintered by the Mo germ layer and embedded and siliconized, a peeling phenomenon of the coating samples could be obviously observed. As shown in Figure 11, the main phases of the exposed area were Ta_5_Si_3_ and TaC after sampling the peeled area for XRD detection. This indicates that at the junction of the carbon diffusion layer and silicon diffusion layer, the coating state was unstable, making it the likely source of crack initiation. The failure may have be caused by Kirkendall holes at the junction of the carbon diffusion layer and the silicon diffusion layer, as described above, due to excessive mutual diffusion of elements. In summary, boride, as the diffusion barrier of the high-temperature oxidation-resistant silicide coating on the tantalum substrate, can better hinder the diffusion of silicon elements and has a better effect of prolonging life.

**Figure 12:**
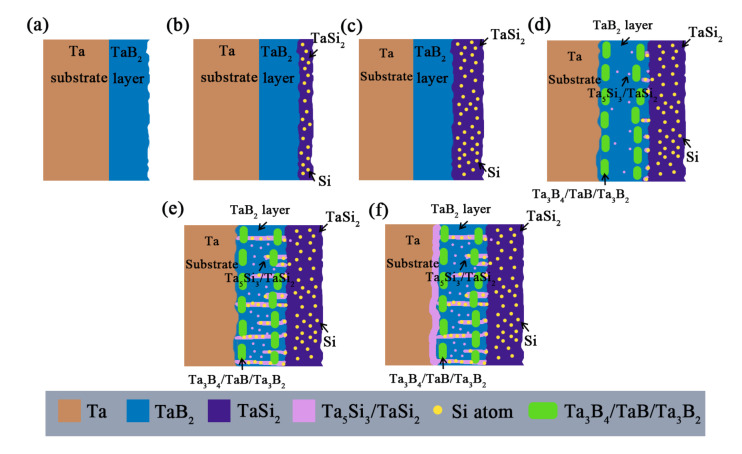
Schematic diagram of the mechanism of the boron diffusion barrier hindering Si diffusion: (**a**) initial state; (**b**) start of siliconizing; (**c**) silicide thickening; (**d**) bidirectional diffusion of boron atoms; (**e**) silicon atoms penetrate through diffusion barriers; (**f**) reaction of silicon atoms with the substrate.

In summary, boride is the type of diffusion barrier most suitable for silicide coating on a Ta substrate. The mechanism of boron diffusion barrier hindering the diffusion of Si is shown in Figure 12. Compared with a Ta substrate, TaB_2_ has a high diffusion energy barrier and lower diffusion coefficient for Si atoms, which can, to some extent, slow down the diffusion of Si atoms into the interior of the coating. In the early stage of diffusion, Si atoms form a layer of TaSi_2_ on the surface of the B diffusion barrier layer, which thickens over time. Subsequently, a few Si atoms diffuse into the boron diffusion layer to form Ta low silicide. B atoms also diffuse in both directions, forming a low-boride portion (Ta_3_B_4_/TaB/Ta_3_B_2_) at the interface on both sides. As diffusion progresses, Si atoms gradually penetrate the B diffusion layer through preferential diffusion channels, bringing in more Si atoms. Finally, Si atoms penetrate the entire boron diffusion barrier layer, enter the substrate, and react with the substrate, causing the diffusion barrier layer to completely fail.

## 4. Conclusions

In conclusion, we explored the influence of the powder ratio and pack cementation temperature on the B diffusion layer on a tantalum substrate through an orthogonal experiment and compared the B diffusion layer with the C diffusion layer from the blocking effect. The results show that the optimal boronizing parameters are a powder ratio of NaF:B:Al_2_O_3_ = 2.5:1:96.5 (wt.%) and a pack cementation temperature of 1050 °C. The silicon diffusion resistance test shows that both B and C diffusion layers have a certain blocking effect on the internal diffusion of silicon (B diffusion layer, 0.48% thickness change rate; C diffusion layer, 21.64% thickness change rate). Compared to the carbide coating, the boride coating has less influence on the outer silicide layer, a lower coating thickening rate, and fewer structural defects, corresponding to better comprehensive blocking performance. Therefore, the results of this work show that it is a better choice to use a boride layer as the diffusion barrier layer of tantalum-based high-temperature oxidation-resistant silicide coatings, as it provides a feasible solution to prevent high-temperature elemental mutual diffusion failure during the application of high-temperature oxidation-resistant silicide coatings on tantalum.

## Figures and Tables

**Table 1:** Orthogonal experimental table of the boronizing process.

Experiment No.	Influencing Factors		
	NaF Content (wt.%)	B Content (wt.%)	Pack Cementation Temperature (°C)
1	2.5	1	1050
2	2.5	2	1100
3	2.5	3	1150
4	5	1	1150
5	5	2	1050
6	5	3	1100
7	7.5	1	1100
8	7.5	2	1150
9	7.5	3	1050

**Table 2:** Range analysis of orthogonal test results of boronized layer thickness.

Experiment No.	Influencing Factors			Thickness of Boriding Layer (μm)
	NaF Content (wt.%)	B Content (wt.%)	Pack Cementation Temperature (°C)	
1	2.5	1	1050	17.9
2	2.5	2	1100	35.8
3	2.5	3	1150	69
4	5	1	1150	28.6
5	5	2	1050	51.3
6	5	3	1100	74.4
7	7.5	1	1100	29.3
8	7.5	2	1150	68.7
9	7.5	3	1050	78.8
K1 (μm)	122.7	75.8	161	–
K2 (μm)	154.3	155.8	143.2	–
K3 (μm)	176.8	222.2	149.6	–
k1 (μm)	40.9	25.26	53.67	–
k2 (μm)	51.43	51.93	47.73	–
k3 (μm)	58.93	74.07	49.87	–
R (μm)	18.03	48.8	5.93	–

**Table 3:** Analysis of variance of orthogonal test results of boriding layer thickness.

Factor	Sum of Squared Deviations	Degrees of Freedom	Mean Sum of Squared Deviations	F Ratio	F Critical Value		Significance
					a = 0.05	a = 0.01	
NaF content (wt.%)	492.40	2	246.20	3.94	19	10.9	–
B content (wt.%)	3582.44	2	1791.22	28.65	19	10.9	Obvious
Pack cementation temperature (°C)	54.20	2	27.10	0.43	19	10.9	–
Deviation	125.04	2	62.52	–	–	–	–

**Table 4:** Composition analysis of original cross-section points of the carbon diffusion layer.

Sampling Point	Elemental Proportions (at.%)
Point 1	C:Ta:Si = 3.9:24.9:71.2
Point 2	C:Ta:Si = 6.5:25.4:68.1
Point 3	C:Ta:Si = 46.9:43.9:9.2
Point 4	C:Ta:Si = 47:45.1:7.9
Point 5	C:Ta:Si = 38.5:58.3:3.2
Point 6	C:Ta:Si = 40.1:54.6:5.3

**Table 5:** Compositional analysis of original cross-section points of the boron diffusion layer.

Pack Cementation Time		Original Thickness	Diffusion Thickness	Thickness Variation	Thickening Rate	Thickening Proportion
0.5 h	Aggregate thickness	40.2	55.4	15.2	37.81	100
	B diffusion layer	13.6	15.3	1.7	12.5	11.18
	Si diffusion layer	26.6	40.1	13.5	50.75	88.82
1 h	Aggregate thickness	46.7	58.8	12.1	25.91	100
	B diffusion layer	19.8	23.7	3.9	19.7	32.23
	Si diffusion layer	26.9	35.1	8.2	30.48	67.77
1.5 h	Aggregate thickness	53.1	64.7	11.6	21.85	100
	B diffusion layer	26.0	42.1	16.1	61.92	67.77
	Si diffusion layer	27.1	22.6	−4.5	−16.61	−38.79

**Table 6:** Compositional analysis of original cross-section points of the carbon diffusion layer.

Sampling Point	Elemental Proportions (at.%)
Point 1	Ta:B:Si = 42.52:54.18:3.3
Point 2	Ta:B;Si = 30.54:68.26:1.2
Point 3	Ta:B:Si = 46.37:53.23:0.4

**Table 7:** Compositional analysis of original cross-section points of the carbon diffusion layer.

Pack Cementation Time		Original Thickness	Diffusion Thickness	Thickness Variation	Thickening Rate	Thickening Proportion
10 min	Aggregate thickness	56.5	60.1	3.6	6.37	100
	C diffusion layer	16.3	11.2	−5.1	−31.29	−141.67
	Si diffusion layer	40.2	48.9	8.7	21.64	241.67
30 min	Aggregate thickness	53.3	64.8	11.5	21.58	100
	C diffusion layer	14.5	13.1	−1.4	−9.66	−12.17
	Transition layer	2.5	3.3	0.8	32	6.96
	Si diffusion layer	36.3	48.4	12.1	33.33	105.22

## Data Availability

The data presented in this study are available upon reasonable request from the corresponding author.
